# Performance of HRP2-based rapid test in children attending the health centre compared to asymptomatic children in the community

**DOI:** 10.1186/1475-2875-13-308

**Published:** 2014-08-09

**Authors:** Gillon Ilombe, Vivi Maketa, Hypolite Muhindo Mavoko, Raquel Inocêncio da Luz, Pascal Lutumba, Jean-Pierre Van geertruyden

**Affiliations:** Clinical Pharmacology Unit, University of Kinshasa, Kinshasa, Democratic Republic of Congo; Department of Tropical Medicine, University of Kinshasa, Kinshasa, Democratic Republic of Congo; Institut National de Recherche Biomédicale, Kinshasa, Democratic Republic of Congo; International Health Unit, Faculty of Medicine, University of Antwerp, Antwerp, Belgium

**Keywords:** Clinical malaria, Asymptomatic malaria, Diagnosis, RDTs

## Abstract

**Background:**

The Democratic Republic of the Congo (DRC) is one of the five countries carrying half of global malaria burden with children 0–5 years old being most at risk. Rapid diagnostic tests (RDTs) are currently routinely used for the detection of Plasmodium infection in health centres and may be a useful tool for population-based survey.

**Methods:**

This study assessed, in a stable transmission zone of Kinshasa, whether a HRP2-based RDT matches the selection criteria of the National Malaria Control Programme (NMCP), DRC and assessed the most relevant fever threshold in this context.

**Results:**

RDTs and microscopy were concordant in 84.3% and 83.4% children in the health centre and at the community level, respectively. The sensitivity was high (>95%), but the specificity was too low and lower in the community (66.9%; 95%CI: 58.5-75.2) compared to the HC (79.4%; 95%CI: 75.7-83.2). The estimated parasitic threshold of 5,414 parasites/μl was with a sensitivity of 63.3% and a specificity of 71.8% not very discriminative, and thus not a threshold.

**Conclusion:**

HRP-based RDT gives a satisfactory proxy to estimate and monitor malaria endemicity, but the low specificity, far below the selection criteria of the NMCP, DRC is problematic for use in a clinical setting.

## Background

Despite all control efforts in the past decade, malaria remains a major public health problem. In 2012, an estimated 207 million malaria episodes occurs worldwide and 80% of the disease burden occurs in sub-Saharan Africa. In 2013, 627,000 deaths were estimated to be attributed to malaria of whom 91% occurred in Africa. 40% of the global mortality rate occurs in only three countries: the Democratic Republic of Congo (DRC), India and Nigeria [1]. Young children are most at risk for severe disease, particularly in high transmission zones, since their immunity has not yet been fully developed [2]. Globally, 86% of malaria related deaths were children below five years of age [1]. According to UNICEF, every minute a child on the African continent dies due to malaria [3].

The DRC has developed a National Malaria Control Programme (NMCP) to monitor the disease and diminish mortality and morbidity. It reported that 97% of the population lives in stable transmission zones putting nearly the entire population at risk for malaria infection. Moreover, mortality in children 0–5 years old is six to seven times higher compared to the general population and they have an average of six to 10 malaria episodes per year [4].

The World Health Organization (WHO) has defined several control strategies, fully or partially adopted by the NMCP, such as prevention of the disease by vector control, management of insecticide resistant vectors, preventive chemotherapy and adequate and timely treatment of cases [5]. The WHO recently abandoned the policy of “presumptive treatment based on clinical signs in children under five years of age, except in cases where biological diagnosis cannot be obtained within two hours and in the case of suspicion of severe malaria” [6]. This signifies that diagnosis must be laboratory-confirmed for all ages prior to administration of anti-malaria drugs and only positive cases should be treated. This change in policy was based on multiple arguments with the main one being that parasitological diagnosis has the highest predictive values and that the proportion of fevers associated to malaria infection is decreasing [7]. However, the debate for the abandonment of presumptive treatment in under-fives is still ongoing. Some still argue that a febrile child under five years of age should be treated presumptively since it is more cost-effective [8]. Moreover, it appears that health workers often do not always take microscopy or Rapid Diagnostic Test (RDT) results into account, resulting in a waste of resources [8].

Parasitological diagnosis of malaria includes either microscopy or RDTs. Microscopy remains the gold standard although its limitations have been demonstrated [9]. RDTs are a quick, simple and cheap alternative and should be used when microscopy is not available [10–12]. At present, there are more than 200 brands on the market and WHO has established selection criteria for the acquisition of RDTs. For the detection of *Plasmodium falciparum*, the panel detection score should be at least 75% at 2,000 parasites/μl; false positive rates should be less than 10% and invalid tests should be less than 5% [13]. The NMCP of the DRC, has adopted these criteria and imposed additional selection criteria such as: the test should be listed by the WHO, sensitivity and specificity should be both >95%, the test should be able to discriminate between *P. falciparum* and other species and finally the test should detect high (>5,000 parasites/μl) and low (≥1 parasite/μl) parasitaemia [14]. Correct diagnosis is essential for effective disease management but the accuracy of RDTs have shown to be variable under field conditions [10–12]. Especially in high-endemic areas, parasitological diagnosis may be challenging because parasitological criteria may not accurately distinguish a symptomatic from an asymptomatic malaria infection. Asymptomatic carriage of parasites occurs frequently in endemic areas and the detection of parasites in a blood film from a febrile individual does not necessarily indicate clinical malaria [15, 16]. Consequently, case definitions for clinical malaria were established indicating that fever together with a parasite density above a specific cut-off is required [16]. The WHO has defined a threshold of 2,000 parasites/μl in a stable transmission zone to be an uncomplicated malaria episode during clinical trials [17]. However, the definition of clinical malaria still varies from one article to another [18, 19]. Moreover, the fever threshold varies according to the intensity, dynamics and seasonality of transmission, and even according to the age of the patients [16, 20]. Therefore, the performance of RDTs to diagnose clinical malaria in highly endemic areas might be hampered due to the high number of asymptomatic carriers of infection.

This study assessed whether an HRP2-based RDT, chosen as the reference by the DRC NMCP, matches the selection criteria of the NMCP in a stable transmission zone of Kinshasa, DRC. In addition, the association between peripheral parasitaemia and the presence of signs and symptoms of malaria was assessed.

## Methods

### Study site

The Health Zone (HZ) of Mont-Ngafula1 is a stable malaria transmission zone situated in the west of Kinshasa, it has 16 Health Areas (HA) and an estimated 219,274 inhabitants. Two HA, Cité Pumbu and Kindele were randomly selected and asymptomatic children were recruited in the community from April to August 2012. Symptomatic children were recruited in the Health Centre (HC) Lisungi from December 2012 to March 2013. The minimal sample size of 280 was estimated taking the prevalence of malaria in the HZ of Mongafula1, 24%, a desired precision set at 5% and confidence level of 95%. Finally, the recruitment ratio was set to 2:1 asymptomatic and symptomatic children.

### Malaria diagnosis

RDTs, SDBioline® Malaria Ag P.f./Pan test (batch nr 05FK60 and 0902065) were kindly donated by the non-profit organization SANRU and they were used according to manufacturer’s instructions. The test can differentiate between *P. falciparum* mono-infection and mixed infections with other *Plasmodium spp*. The test was considered positive by the appearance of the antigen and control lines in their respective windows. Corresponding to the positions, tests were read as positive to *P. falciparum* infection, positive to other *Plasmodium spp*. or mixed infections. Negative results were recorded in case the control band was visible and results were invalid when the control band was not visible. In case of an invalid result, the RDT was repeated. To ensure the validity of the results, RDTs were read within a timeframe of maximal 15 minutes by two independent laboratory technicians.

For microscopic diagnosis, thick and thin blood smears were collected on the same slide and stained with 10% Giemsa for 10 minutes. Thin smears were fixed with methanol prior to staining. Slides were examined by two independent laboratory technicians. The parasite density was estimated assuming 8,000 white blood cells/μl [9]. In case of >15% discrepancy, a third laboratory technician’s judgment was required.

### Statistical analysis

Data were double entered and validated in Epi Info 3.5.1. and analysed using Stata 11.1 (Stata Corp, Lakeway, College Station, Texas, USA). Age, sex and history of fever were also recorded. History of fever was reported by the parents as the presence of fever in the past 24 h. For data with skewed distribution, the median and its interquartile range (IQR) are reported. Due to a high number of negative patients in the community, children with a parasite density of 0 were excluded to calculate the median. A log transformation of the data was performed to compare the mean parasite density with the unpaired t-test. For binary data, the chi^2^ test was applied. Results were considered statistically significant when p ≤ 0.05 or when 95% Confidence Intervals (CI) are not overlapping. The sensitivity and specificity of the RDT’s were calculated in each study group using the thick blood smears (TBS) as the reference. The optimal parasite density to discriminate symptomatic from asymptomatic children or feverish form non-feverish children were estimated with a ROC analysis. The Kappa value was calculated to measure the concordance between RDT results and fever.

### Ethical considerations

Ethical approval for this study was provided by Ethical committee of the School of Public Health, Kinshasa University, DRC. Participation was entirely voluntary and written informed consent was obtained from the parents or legal guardians of the children. Confidentiality is guaranteed by restricting access to the data. Medication was given to the children when their biological profile indicated the need of therapeutic care.

## Results

**Figure 1:**
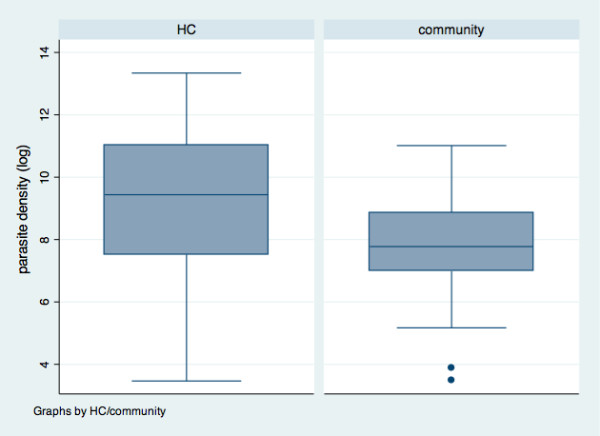
**Distribution of the parasite density in asymptomatic and symptomatic children in Kinshasa, RDC, 2012–3.**

A total of 872 children were recruited into the study, 592 were recruited from the community while 280 children presenting fever (body temperature ≥37.5°C) were recruited in the Health Centre (HC). Median age was 35.1 months old (range: 0.7-59.9) and 53.8% of the children were male (Table 1). For all the children in the HC, history of fever was reported whereas 1.9% of the children in the community had a history of fever. In the study population, the prevalence of malaria infection was 34.4% according to microscopy, whereas it was 48.6% when RDT was used. Symptomatic children had higher prevalence of malaria infection both with microscopy as RDT than asymptomatic children (Table 1). Children in the HC presented a mean parasite density ± standard deviation (SD) that was significantly higher (geometric mean: 10 807.4; SD: 8.2 parasites/μl) compared to the children in the community (geometric mean 2 788.4; SD: 4.2 parasites/μl) (p < 0.001) (Figure 1). However, the lowest parasite densities were found in children attending the HC.

**Table 1:** **Characteristics asymptomatic children in the community** ***versus*** **symptomatic children in the Health Centre in Kinshasa, RDC, 2012-3**

Variables	All (N = 872)		Health Centre (N = 280)		Community (N = 592)	
	n	%(95%CI)	n	%(95%CI)	n	%(95%CI)
**Sex**						
M	469	53.8 (50.5-57.1)	147	52.5 (46.5-58.5)	322	54.4 (50.3-58.4)
F	403	46.2 (42.9-49.6)	133	47.5 (41.5-53.5)	270	45.6 (41.6-49.7)
**History of fever**	291	32.6 (30.3-36.6)	280	100 (100–100)	11	1.9 (1.0-3.4)
**Positive blood smear**	300	34.4 (31.3-37.7)	156	55.7 (49.7-61.6)	144	24.3 (21.0-28.0)
**Parasite density ≥2,000 troph/μl**	190	21.8 (19.1-24.7)	114	40.7 (34.9-46.7)	76	12.8 (10.3-15.9)
**RDT +**	424	48.6 (45.2-52.0)	194	69.3 (63.5-74.6)	203	38.9 (34.9-42.9)
***P. falciparum***	142	16.3 (13.9-18.9)	40	14.3 (10.4-18.9)	102	17.2 (14.3-20.6)
Mixed infection	282	32.3 (29.3-35.6)	154	55.0 (49.0-60.9)	128	21.6 (18.4-25.2)

**Figure 2:**
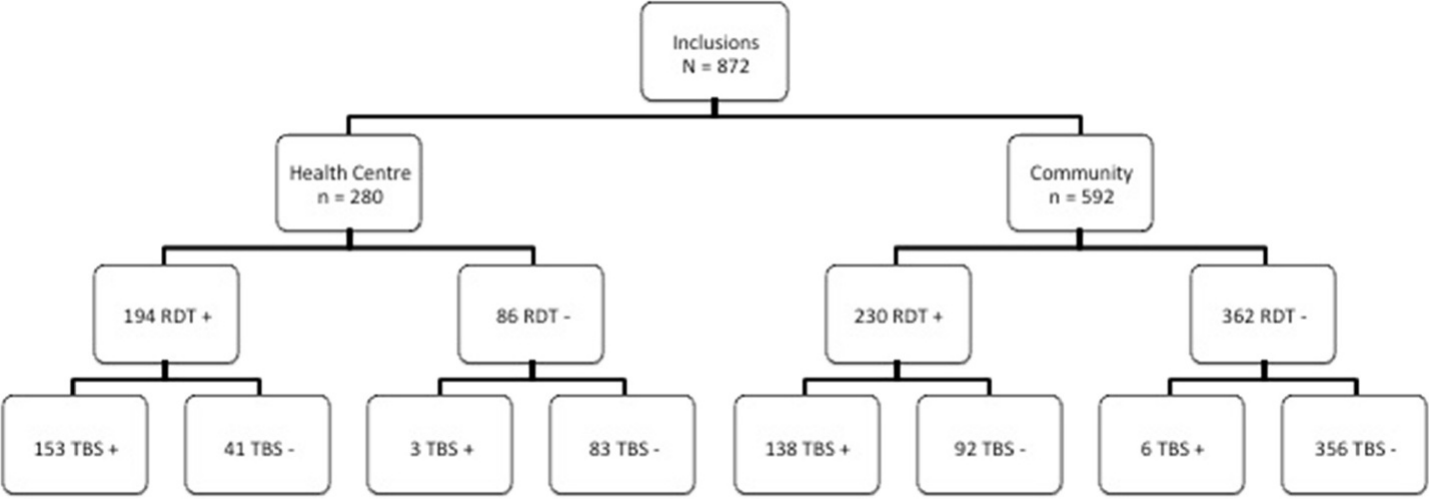
**Flow chart of RDT and microscopy result in asymptomatic and symptomatic children in Kinshasa, RDC, 2012–3.**

Figure 2 presents the flow chart of test results. In both settings, the concordance between RDT and microscopy were similar with 84.3% concordant results in the HC and 83.4% concordant results at the community level (p = 0.8). Sensitivity was more than 95% in both settings, but the specificity was lower in the community (66.9%; 95%CI: 58.5-75.2) compared to the HC (79,4%; 95%CI: 75.7-83.2) although it was not significant (Table 2). The sensitivity of the RDT increased with increasing parasite density in both settings and there were no significant differences between the two groups. However, the sensitivity was more variable in the community. The positive predictive value (PVV) was higher in the HC (60%) (Figure 2, Table 2). The rate of false positives was 21.1% in the HC compared to 40.0% in the community (p < 0.001). The negative predictive value (NPV) was high in both settings: 98.3% (95%CI: 97.0-99.6) *versus* 96.5% (95%CI: 92.5-100) in the community and HC respectively. Table 3 shows the RDT results according to the parasite densities found by microscopy showing false positive and false negative results for each parasite density.

**Figure 3:**
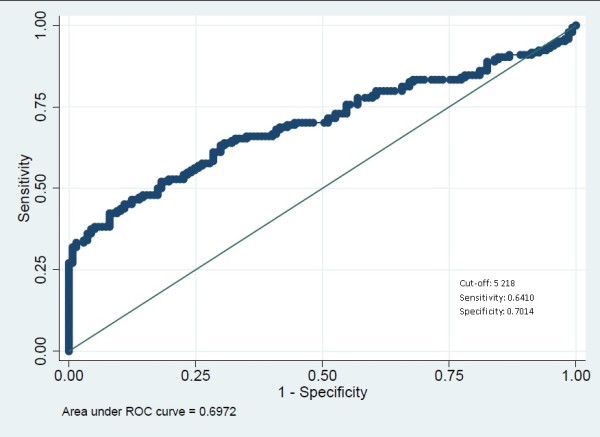
**ROC curve to assess symptomatic parasitaemic threshold in children in Kinshasa, RDC, 2012–3.**

**Figure 4:**
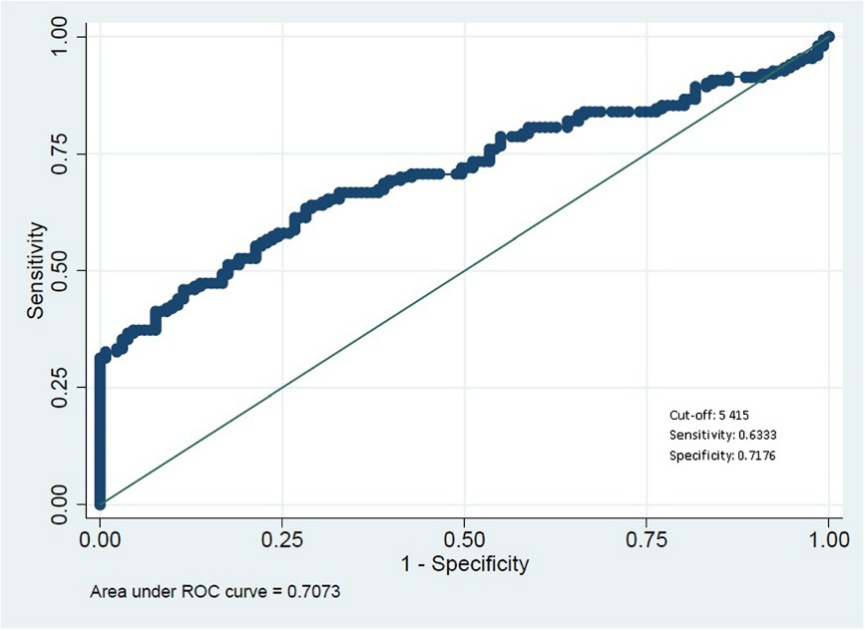
**ROC curve to assess the fever parasitaemia threshold in children in Kinshasa, RDC, 2012–3.**

The children were categorized as symptomatic when attending the HC and at the community level they were categorized as asymptomatic. ROC analysis estimated 5,218 parasites/μl to be the discriminating cut-off. This cut off point had a sensitivity and specificity of 64% and 70% respectively, and the area under the curve was 0.70 (Figure 3). Some children in the community presented fever although medical assistance was not sought. History of fever was consequently included into a model to enhance the discriminatory power of the cut-off value. The cut-off to distinguish children with history of fever from those without was 5414 parasites/μl. This cut-off point was similar to the previous one with a sensitivity and specificity of 63% and 70% respectively with the area under the curve being 0.70 (Figure 4).

**Table 2:** **Performance of RDT with microscopy as reference in asymptomatic and symptomatic children in Kinshasa, RDC, 2012-3**

	Sensitivity %(95%CI)	Specificity %(95%CI)	PPV %(95%CI)	NPV %(95%CI)
**Community**	95.8 (92.5-99.1)	79.4 (75.7-83.2)	60.0 (53.6-66.3)	98.3 (97.0-99.6)
**Health Centre**	98.0 (95.9-100)	66.9 (58.5-75.2)	78.8 (73.0- 84.6)	96.5 (92.5-100)

**Table 3:** **RDT results stratified by parasite density in asymptomatic and symptomatic children in Kinshasa, RDC, 2012-3**

Parasite density (trophozoites/μl)	RDT results			
	HC		Community	
	Neg.	Pos.	Neg.	Pos.
Negative	83	41	356	92
1-1,000	2	20	3	29
1,001-2,000	1	19	0	37
2,001-3,000	0	6	1	10
3,002-4,000	0	5	0	9
4,001-5,000	0	2	2	8
>5,000	0	101	0	45

## Discussion

Accurate diagnosis of malaria infection is an important matter since in high and stable transmission zones, a high proportion of the population are asymptomatic carriers of infection. This proportion of the population might also need effective anti-malarial treatment because spontaneous resolution of untreated infections is relatively rare [15]. The overall concordance between microscopy and RDT was quite good (>83%) in both settings (Figure 2). False negative results were low in both settings and occurred mostly in low parasite densities (<2,000 parasites/μl) in the HC, at the community level it also occurred in higher parasite densities. The sensitivity of the RDT increased was correlated with parasite density confirming former reports [20, 21]. Moreover, sensitivity was high in both settings and thus met the criteria set by the NMCP. However, at the community level, three false negatives were at low parasite densities, but three false negatives were also found at higher parasite density (Table 3). Testing in the community required going into the field, and with the hot climate of Kinshasa, poor conservation may have affected the results [22].

The vast majority of discordant results were due to the high rate of false positive results. In the HC it was 21.1% and it was unacceptable high at the community level (40.0%) thus RDT systematically overestimated the prevalence which may be problematic (Table 1) as it leads to overtreatment of children and increases the drug use and pressure [22]. Moreover, in the HC other causes of fever may be overlooked and or correct diagnosis delayed while treating for malaria. It is well known that HRP-based tests may remain positive for over a month after appropriate treatment due to the persistence of HRP2 antigen [20, 23]. On the other hand, the high number of false positive results could be explained by sub-microscopic infections. In several malaria-endemic areas, PCR measurements have demonstrated that microscopy can miss many cases with a low-density parasitaemia, showing many apparent false-positive RDT results [15]. This phenomenon is, however, more pronounced in low transmission zones. Nevertheless, PCR consistently detects a higher prevalence of infection (on average 49.2%) compared to microscopy [24]. It was not assessed in this study but PCR analysis of the samples may substantially reduce the number of false positive results. Consequently, it influences the specificity found in this study, which was very low, especially at the HC (66.9%) (Table 2). Both phenomena, persistence of HRP2 antigen and sub-microscopic infection, might partially explain the high rate of false positive results found in this study. Some children may have had either proper treatment for a previous infection or received auto-medication from their parents. Others could simply have sub-microscopic infections prior to manifestation of parasites in the blood smear. This was unfortunately not assessed in this study.

The NMCP of the DRC endorses the use of RDTs in Health Centres and might extend its use to monitor malaria prevalence in the general population to enhance control efforts. Based on the formulated criteria [14], the NMCP selected SD Bioline® as the reference. However, it was not able to fulfil all criteria imposed by the NMCP. The sensitivity passed the threshold but specificity stayed far below the required 95%, with 21.1% and 40.0% false positive results in the HC and community respectively. These values stay even below the less stringent the WHO threshold. The PPV was higher in the HC compared to the community, which is logic since the malaria prevalence was higher in the HC. The RDT discriminated successfully *P. falciparum* from other species (Table 1). Finally, the test should also be able to detect high (>5,000 parasites/μl) and low (≥1 parasite/μl) parasite densities [14]. However, it is in reality very difficult to comply to this criterion. However, the test was able to detect very low parasite densities despite a number of false negative results. On the other hand, the question remains if it is clinically relevant to detect such a low parasite density in the community where the children do not present clinical symptoms. Since spontaneous resolution of untreated infections is rare, one might consider treating the malaria infection [15].

Clinical diagnosis of malaria illness also represents a challenge because *P. falciparum* infection leads to non-specific signs and symptoms and the majority of the population in endemic areas remain disease-free despite high parasitaemia [18]. Nevertheless, a relationship between fever and parasite density exists. It has led to case definitions for clinical malaria based on fever together with a parasite density above a specific cut-off [16]. In this study, the median parasite density was significantly higher in symptomatic children (HC) compared to asymptomatic children (Figure 1). The discriminatory parasitic density was found to be 5,218 parasites/μl (Figure 3). However, the surface under the ROC curve indicated that parasitic density as such has low discriminative power to distinguish either feverish or symptomatic children. This threshold of more than 5,000 parasites/μl is way above the cut-off of 3,000 parasites/μl reported by Mulumba *et al.* [25]. This could be due to a different transmission level as they compiled their results in a high and low transmission zone influencing the proportion of asymptomatic carriage of infection [18, 19]. On the other hand, other authors working in the high transmission zone of Kinshasa have found a higher threshold than presented here. The parasite density threshold seems thus very variable and not discriminant as it depends on various factors. Moreover, it may change over time when effective control measures have been taken by the government [26]. This constitutes a real challenge for research on malaria illness because a clear definition of clinical malaria remains inexistent. In addition, the parasite density found in the HC had a large range. Children with low parasite densities were also presenting fever while children with high parasite densities were asymptomatic in the community. The individual variation is thus too high to adequately define clinical malaria. This has serious implications for disease management of malaria. Moreover, RDTs do not provide any estimate of the parasite density. Since no satisfactory cut-off value could be found, and asymptomatic infection of *P. falciparum* is not a harmless condition, it would be recommended to treat all RDT positive cases with the recommended anti-malaria drugs. However, due to the relatively high number of false positive RDT results, clinicians should still check whether there may be co-infection with other organisms. Because when a false positive results influences antibiotic prescription, it endangers the health status or even the life of the child [27]. Clinicians should on the other hand adhere to RDT negative results, refrain from anti-malaria treatment and look for other pathologies that can explain the fever.

A limitation of the study was that sub-microscopic infections were not assessed with a more sensitive PCR assay. It may significantly reduce the number of false positive RDT results. History of fever is a subjective indicator reported by the parents. Reporting history of fever is a very useful and frequently used in several studies where presumptive malaria is evaluated [28, 29]. Presumptive treatment has been recently abandoned as they could favour the appearance of drug resistant strains and a delay of proper treatment for the child [30]. In the community, 11 cases (1.9%) were found be feverish. This small proportion had no impact on the study results.

## Conclusion

HRP-based RDTs performed quite similarly in both the HC and overestimated the malaria prevalence systematically by almost 15% and may be a satisfactory proxy to estimate and monitor malaria endemicity but the low sensitivity, far below the selection criteria of the NMCP, DRC is problematic for use in a clinical setting and has to be further explored. The parasite density threshold seemed very variable and not discriminant and its usefulness is questionable.

## Abbreviations

DRC: Democratic Republic of the Congo
HA: Health Area
HC: Health Centre
HZ: Health Zone
NMCP: National Malaria Control Programme
NPV: Negative predictive value
PPV: Positive predictive value
RDT: Rapid Diagnostic Test
SD: Standard deviation.
